# Stem cell factor in allergic diseases: mechanisms of type 2 immunity and therapeutic opportunities

**DOI:** 10.3389/fimmu.2026.2009363

**Published:** 2026-10-29

**Authors:** Ramon Ocadiz-Ruiz, Sarita Montaño, Nicholas W. Lukacs, Wendy Fonseca

**Affiliations:** 1 Department of Pathology, University of Michigan Medical School, Ann Arbor, MI, United States; 2 Mary H. Weiser Food Allergy Center, University of Michigan, Ann Arbor, MI, United States

**Keywords:** allergy, asthma, c-kit, ILC2, ILCP, SCF, type 2 inflammation

## Abstract

Stem cell factor (SCF) and its receptor c-Kit regulate the development, survival, tissue localization, and activation of several immune populations central to allergic disease. This review examines how the SCF220 and SCF248 isoforms shape c-Kit signaling in mast cells, group 2 innate lymphoid cells, eosinophils, dendritic cells, and basophils; how stromal and epithelial sources of SCF sustain allergic inflammation; and how this pathway contributes to disease severity and tissue remodeling. We also discuss the therapeutic potential and limitations of targeting SCF, or specific SCF isoforms, in allergic disease.

## 1 Introduction

Allergic diseases, including asthma, allergic rhinitis, chronic rhinosinusitis, atopic dermatitis (AD), eosinophilic esophagitis (EoE), and food allergy, are major contributors to global morbidity and collectively impose substantial healthcare costs (1). Although clinically diverse, these disorders share several key features, including epithelial barrier dysfunction, immune-cell infiltration, tissue remodeling, microbiome alterations, and type 2 inflammation (1, 2). Allergens, respiratory infections, and environmental irritants stimulate epithelial release of alarmins (thymic stromal lymphopoietin (TSLP), interleukin (IL)-25, and IL-33) and chemokines, including CCL2, CCL5 (RANTES), CCL11 (eotaxin), and CXCL8 (interleukin-8). These mediators promote the activation and/or recruitment of various immune cells involved in type 2 inflammation, including group 2 innate lymphoid cells (ILC2s), dendritic cells, mast cells (MCs), basophils, eosinophils, and T cells, leading to production of IL-4, IL-5, IL-9, and IL-13 (1–4).

Type 2 cytokines including IL-4, IL-5, IL-9, and IL-13 coordinate the key effector mechanisms of allergic inflammation. IL-4 promotes Th2 differentiation, IgE class switching, and expression of VLA4 and VCAM-1 that support T-cell and eosinophil trafficking (5). IL-5 promotes eosinophil maturation and recruitment; IL-9 supports eosinophilic inflammation, mast-cell growth, mucus hypersecretion, and airway hyperresponsiveness (AHR); and IL-13 promotes IgE-producing B-cell proliferation, goblet-cell hyperplasia, mucus production, AHR, and epithelial tight-junction (TJ) disruption (6, 7).

Several therapies have been developed to target type 2 inflammatory pathways, including inhibitors TSLP (Tezepelumab, AZD8630), IL-33 (Itepekimab, Tozorakimab), ST2 (Astegolimab), IL4Ra (Dupilumab), IL-13 (Tralokinumab), IL-13Rα1 (Eblasakimab), and IL-5 (Mepolizumab), as well as JAK (JAKi), BTK (BTK), tryptase (MTPS9579A mAb), Siglec-8 (lirentelimab), c-Kit (barzolvolimab), and IgE (omalizumab) (1). As well as signaling pathways in type 2 inflammation, such as JAKs, a family of intracellular tyrosine kinases that transduce cytokine-mediated signals via the JAK–STAT pathway; Janus kinase inhibitors (JAKi) or Bruton’s tyrosine kinase is an intracellular molecule involved in the signaling pathway downstream of mast cell and basophil IgE–FcϵRI cross-linking and in B cell receptor (BCR) activation (BTK) inhibitors) (1). Persistent disease in some patients despite these therapies underscores the need to investigate upstream pathways that simultaneously regulate multiple allergic effector cells.

Stem cell factor (SCF) is produced by bone marrow stromal cells, endothelial cells, keratinocytes, adipocytes, fibroblasts, and other structural cells. Through c-Kit, SCF regulates the development, survival, localization, and activation of hematopoietic stem cells and allergic effector populations, including mast cells, eosinophils, dendritic cells, and ILCs. Two major isoforms, SCF220 and SCF248, differ in processing and expression patterns (8, 9). Because c-Kit is expressed by several key drivers of allergic inflammation, the SCF-c-Kit axis may sustain disease through coordinated effects across cell types and represents a potential therapeutic target. This review focuses on SCF isoform biology, c-Kit signaling, immune cell-specific mechanisms, evidence from allergic diseases, and therapeutic opportunities.

## 2 SCF structure, isoforms, and biological functions

SCF, also known as c-Kit ligand, mast cell growth factor, or steel factor, is encoded by c-KITLG in humans and *C-Kitl* (the Steel locus) in mice. The human gene is located on chromosome 12, whereas the murine Steel locus is on chromosome 10. SCF signals through the receptor tyrosine kinase c-Kit (c-Kit/CD117) (8, 10–12).

SCF forms a noncovalent homodimer that binds two c-Kit molecules to generate a 2:2 signaling complex. Structural studies show that c-Kit wraps around the SCF dimer through its first three immunoglobulin-like domains, while additional receptor-receptor contacts involving membrane-proximal domains stabilize the active dimer. Thus, c-Kit activation depends on both ligand-mediated assembly and direct receptor interactions, followed by kinase-domain trans-autophosphorylation (13, 14).

Two major SCF isoforms are generated by alternative splicing in humans and mice: SCF248 and SCF220 (**Figure 1**). Both are synthesized as transmembrane proteins and share the same C-KIT-binding extracellular region. SCF248 contains the major proteolytic cleavage site encoded by exon 6 and is efficiently processed to release soluble SCF. In contrast, SCF220 lacks this site and is retained predominantly on the cell surface, although limited cleavage may occur at alternative sites. SCF is biologically active as a dimer. Because soluble SCF is generally monomeric, it induces little or no c-Kit activation, whereas dimerized membrane-bound SCF promotes robust c-Kit activation and downstream receptor signaling (15–17) (**Figure 1**).

**Figure 1:**
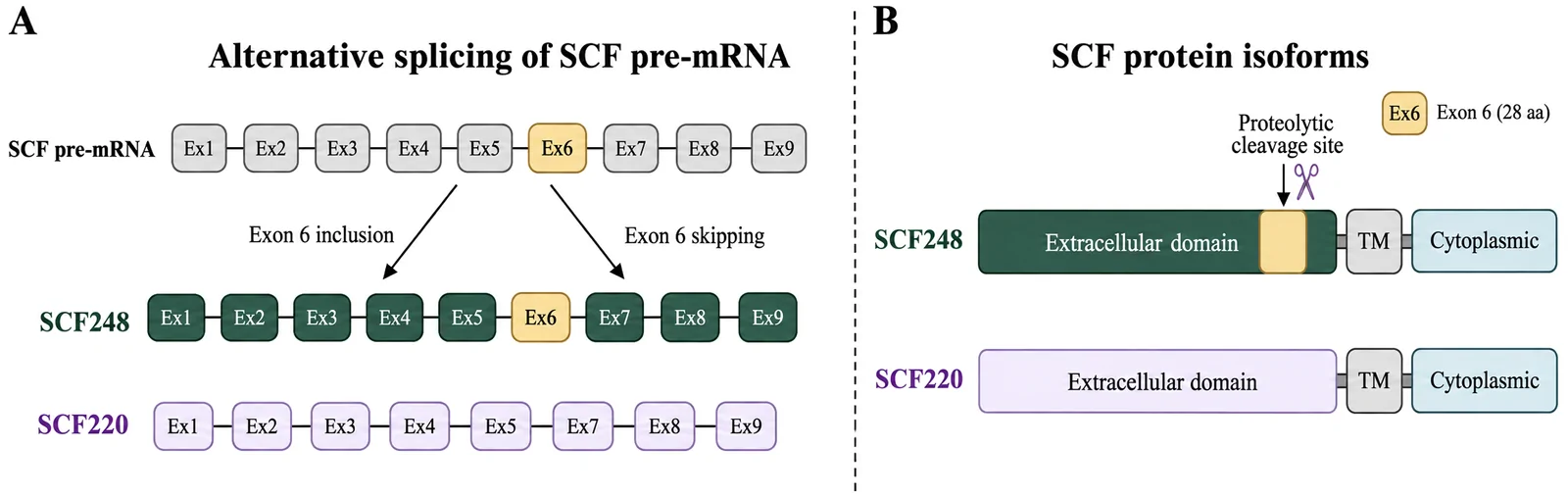
SCF isoforms. There are two SCF isoforms, SCF220 and SCF248. **(A)** Alternative splicing of SCF pre-mRNA generates transcripts encoding SCF248 through exon 6 inclusion or SCF220 through exon 6 skipping. **(B)** Both isoforms contain extracellular, transmembrane (TM), and cytoplasmic domains. SCF248 contains the exon 6-encoded region encompassing the major proteolytic cleavage site that enables soluble SCF release. when SCF248 is cleavage, it releases soluble SCF, a monomeric form that cannot activate c-Kit but can be detected in serum. However, in its membrane form, SCF isoforms can efficiently crosslink and, as a membrane-bound homodimer, can bind to and activate its receptor, c- KIT; their distinct processing and expression patterns may produce differences in signaling duration and biological context. Adapted from Dehbashi et al., “Comparative genomics of human stem cell factor (SCF)” (PMID: 28447043), and redrawn and revised using OpenAI’s image-generation platform in ChatGPT.

Membrane-associated SCF is produced by bone marrow stromal cells and multiple structural-cell populations, including endothelial cells, keratinocytes, adipocytes, and fibroblasts (18–23). Studies using Sl/Sld mice and isoform-reconstitution models established that membrane SCF is required for normal hematopoiesis and mast-cell development. SCF220 more effectively supports growth and erythroid homeostasis, whereas selective loss of SCF248 has a stronger effect on peripheral myeloid populations, particularly mast cells. These findings support distinct, nonredundant functions for the two splice variants. Although SCF248 is critical for myeloid cell development and SCF220 for erythropoiesis, these predominant roles do not exclude either variant from contributing to other biological pathways (24–26).

## 3 SCF-c-kit signaling

c-Kit (CD117) belongs to the type III receptor tyrosine kinase family, along with PDGFRA/PDGFRB, CSF1R, and FLT3 (27, 28). The receptor contains five extracellular immunoglobulin-like domains, a single transmembrane segment, a juxtamembrane regulatory region, and a split cytoplasmic tyrosine kinase domain (27, 28). c-Kit is expressed by hematopoietic stem cells, melanocytes, progenitor cells, mast cells, eosinophils, dendritic cells, basophils, and innate lymphoid cells. SCF-c-Kit signaling regulates survival, proliferation, adhesion, migration, differentiation, and activation in a cell- and context-dependent manner (8, 29, 30).

Key phosphorylated tyrosines include Y568/Y570, which recruit SRC-family kinases; Y703, which recruits GRB2; and Y721, which binds the p85 regulatory subunit of PI3K. These interactions activate RAS-RAF-MEK-ERK, PI3K-AKT-mTOR, SRC-family, PLCγ, and JAK/STAT-associated signaling networks that regulate survival, proliferation, migration, adhesion, and mediator production (31–33). The relative strength and duration of these pathways vary with ligand form, receptor abundance, and cell type.

## 4 SCF expression in allergic inflammation

SCF248 expression increases in several allergic tissues and experimental models, with stromal cells, fibroblasts, epithelial cells, endothelial cells, and myofibroblasts serving as important sources (23, 34). Human studies report increased airway SCF expression and elevated circulating soluble SCF in asthma, with serum levels associated with disease severity. SCF248 isoform contains a proteolytic cleavage site that enables the release of soluble SCF. Thus, elevated soluble SCF in human asthma is consistent with increased SCF248 expression and proteolytic processing, supporting a potential role for this isoform in disease. (35–37). Collectively, these findings indicate that SCF248 plays an important role in allergic responses.

In chronic allergic-airway models, SCF248 neutralization or genetic disruption of SCF/c-C-Kit signaling reduces mast cells, eosinophils, ILC2s, type 2 cytokines, mucus-associated responses, and disease severity (23, 38, 39). Allergic inflammation also increases SCF248 in bone marrow, where it is associated with expansion of ILC progenitors and contributes to systemic ILC2 generation. Selective blockade of SCF248 reduces bone-marrow ILCp, circulating and pulmonary ILC2. These data support a stromal-SCF/c-C-Kitcircuit that acts both locally and systemically during chronic allergy (23, 39). Our group has demonstrated that IL-13, IL-1β, and IL-33 induce SCF248 over expression in mesenchymal cells, with IL-13 producing the strongest response in the tested *in vitro* system (23). SCF248 was selectively increased in bone marrow during chronic allergic inflammation, whereas SCF220 remained comparatively stable (23).

## 5 Role of SCF in the activation and regulation of c-C-KIT^+^ allergic effector cells

### 5.1 Group 2 innate lymphoid cells

Innate lymphoid cells play important roles in host defense against pathogens, and in maintaining tissue homeostasis. c-Kit (CD117) is expressed by innate lymphoid cell progenitors and retained at variable levels across mature ILC populations, with ILC2s displaying uniquely heterogeneous expression that marks functionally distinct subpopulations (40). Among these populations, ILC2s are potent producers of the type 2 cytokines IL-5 and IL-13, which are key mediators of allergic inflammation, in response to tissue alarmins such as IL-33 and thymic stromal lymphopoietin (TSLP). Studies in the airways and other barrier tissues have shown that ILC2-derived IL-5 and IL-13 promote eosinophil recruitment and survival and modulate mast-cell activity, thereby linking early alarmin-driven ILC2 activation to downstream effector responses in allergic disease (41–43). Clinical studies confirm that ILC2s are increased in the tissue of patients with food allergy, atopic dermatitis, eosinophilic esophagitis, chronic rhinosinusitis, allergic asthma, and allergic rhinitis (44, 45–54).

SCF/c-Kit signaling supports ILC2 maturation, activation, accumulation, and effector fitness signal at baseline and during allergic inflammation (23). In a murine model of chronic allergic asthma, SCF248 blockade and impaired c-Kit signaling reduce c-Kit^+^ ILC2 accumulation and type 2 cytokine production (23, 39). Recent studies found that SCF deficiency altered hematopoietic ILC2 cells transcriptional programs, including reduced *Il4, Stat5b, Akt2, Sh2b1, Gata3, Icos, and Rora*; SCF248-rich OP9-DL1 cultures increased *GATA3, ID2, ICOS, CD90, c-Kit, Il4*, and *Il13* and increased mature ILC2 numbers. *In vivo*, inducible SCF deletion or SCF248 blockade reduced bone-marrow ILCp, circulating ILC2, pulmonary ILC2, and allergic inflammation (23). These findings identify SCF248 as an amplifier of ILC2 maturation and mucosal allergic responses, while direct lineage-tracing evidence for clonal expansion remains limited. Together, these studies support a model in which constitutive SCF220 contributes to baseline c-Kit signaling, whereas inducible SCF248 amplifies ILC2 maturation and activation during chronic allergic inflammation. SCF enhances IL-25R (IL-17RB) and IL-9R expression and can increase cytokine responses to IL-25 and IL-9, while the fungus allergy model demonstrates that SCF deletion or SCF248 blockade limits ILC2 responses even in an IL-33-rich environment (23, 39). Moreover, SCF248 promoted the acquisition of ILC2-associated transcription factors, surface markers, and effector cytokines during *in vitro* ILCp differentiation, supporting a role for SCF/c-Kit signaling in ILC progenitor expansion, phenotypic programming, and maturation into ILC2s (23, 39).

The developmental pathways that give rise to ILC2s are not fully defined; however, human ILC2s are thought to originate from ILC progenitors with either unilineage or multilineage potential. ILC progenitors are characterized by expression of c-KIT, which is broadly associated with hematopoietic progenitor populations. c-Kit expression is also retained by a subset of ILC2s (45, 55). Recent evidence indicates that c-KIT^+^ ILC2s exhibit an ILC progenitor-like epigenomic profile that is consistent with their enhanced phenotypic and functional plasticity. Notably, this c-KIT^+^ state has been associated with susceptibility to immune-mediated diseases such as asthma, supporting the concept that c-KIT expression identifies a developmentally flexible ILC2 population rather than merely serving as a lineage marker (56). These findings also raise the possibility that signals acting through c-KIT, including SCF, may influence the maintenance or functional programming of this plastic ILC2 state; however, the direct contribution of SCF-c-Kit signaling remains to be established.

Human ILC2s are heterogeneous for c-Kit expression, and SCF costimulation can enhance IL-5 and IL-13 production in c-Kit^+^ ILC2s subsets without establishing that SCF determines their plasticity (40). In response to changes in the local cytokine environment, human ILC2s can acquire ILC1-like or ILC3-like phenotypes (40, 57, 58). This plasticity appears particularly relevant in chronic inflammatory diseases of the airways (39, 59, 60) and skin (59, 61), with c-Kit^+^ ILC2s proposed to exhibit greater phenotypic flexibility (40). Beyond driving classical type 2 inflammation, c-Kit^+^ ILC2s exhibit profound functional plasticity, making them capable of trans-differentiating into an ILC3-like phenotype under chronic inflammatory conditions. Notably, while mature ILC2s are the primary sources of IL-5 and IL-13 (**Figure 2**), microenvironmental cues drive these c-Kit^+^ cells to undergo plastic conversion. They transition into IL-17A-producing ILC3-like cells, thereby contributing directly to severe neutrophilic infiltration during asthma exacerbations (40, 62–64). The precise molecular mechanisms governing this functional reprogramming remain incompletely understood, but they underscore SCF/c-Kit signaling as a broad therapeutic target capable of modulating both canonical type 2 and converted type 3 innate pathways (40, 62–65). However, the molecular mechanisms underlying c-Kit^+^ ILC2 plasticity remain incompletely understood. Recent evidence indicates that c-Kit expression in human ILC2s is dynamically regulated by the cytokine environment. c-Kit^+^ ILC2s exhibit a precursor-like epigenetic landscape resembling that of ILC progenitors, consistent with enhanced cellular plasticity. Although these findings do not establish that IL-2 and IL-7 directly generate this epigenetic landscape, they suggest that tissue-associated c-Kit phenotypes may be shaped by the local inflammatory microenvironment (40, 66).

**Figure 2:**
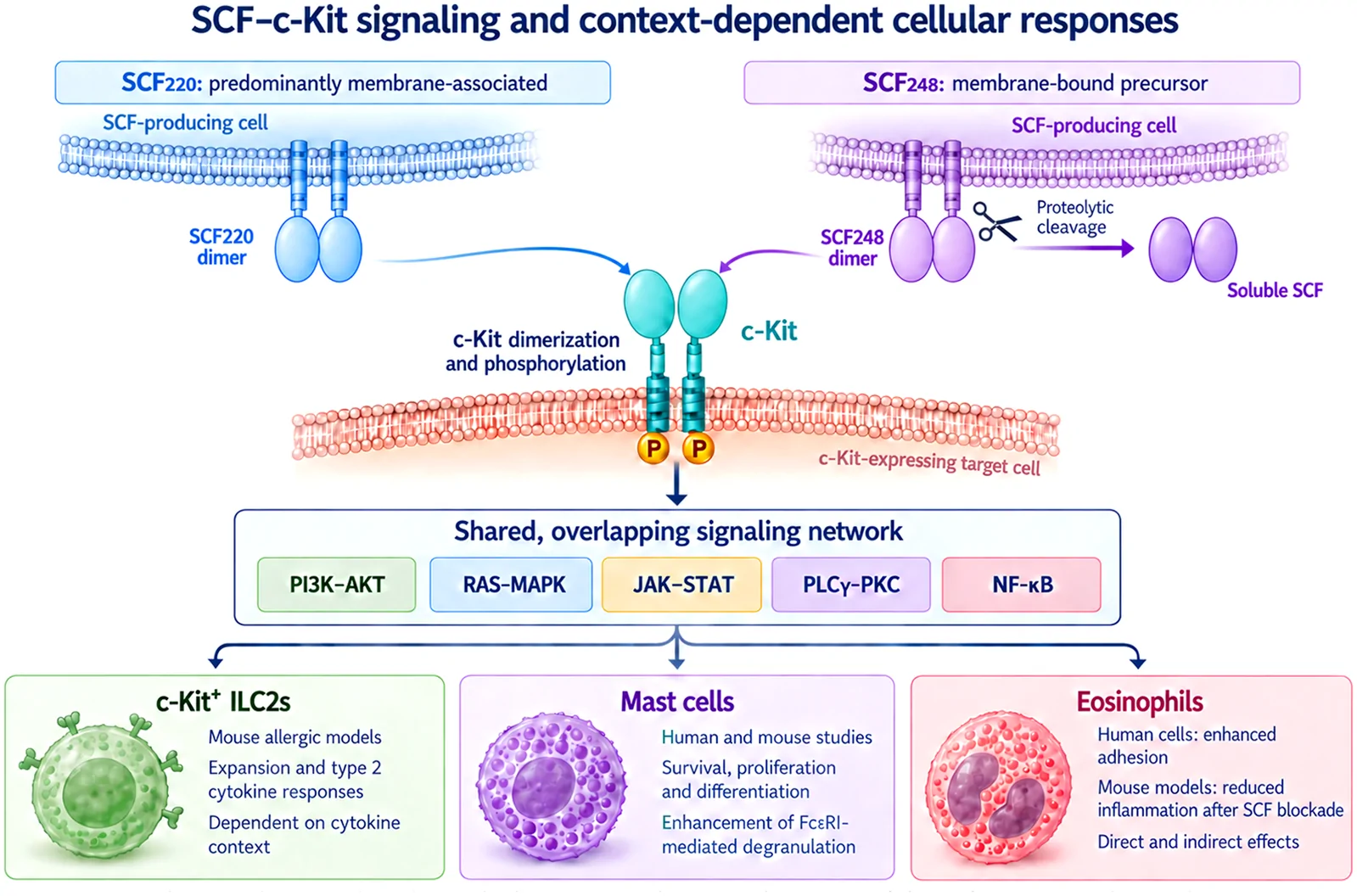
SCF/c-KIT signaling and cellular responses. SCF220 is predominantly membrane-associated, whereas SCF248 is initially membrane-bound and can undergo proteolytic cleavage to release soluble SCF. The schematic emphasizes membrane-associated SCF engagement of c-Kit, promoting receptor dimerization, phosphorylation, and downstream signaling through overlapping PI3K-AKT, RAS-MAPK, JAK-STAT, PLCγ-PKC, and NF-κB pathways. Cellular responses include expansion and type 2 cytokine responses in c-Kit-expressing ILC2s; mast-cell survival, proliferation, differentiation, and enhancement of FcϵRI-mediated degranulation; and enhanced eosinophil adhesion. ILC2 responses primarily reflect mouse allergic models and depend on cytokine context. Eosinophil adhesion is supported by human cell studies, whereas reduced eosinophilic inflammation following SCF blockade in mouse models may involve direct and indirect effects. Mast-cell responses summarize human and mouse studies. Pathway contributions vary with cell type, species, and experimental conditions.

Given the abundant expression of SCF within hematopoietic compartments, SCF-c-Kit signaling may provide an important niche signal that supports c-Kit-expressing ILC progenitors regulates their phenotype, maintenance, and differentiation into mature ILC2s.

### 5.2 Mast cells

Mast cells show exceptional dependence on SCF/c-Kit signaling for tissue survival, proliferation, migration, and terminal maturation. Loss of function mutations in c-Kit or c-Kitl produce profound tissue mast-cell deficiency, and administration of SCF expands mast-cell populations *in vivo* (40, 67, 68).

SCF is widely regarded as the main growth factor for mast cells, controlling nearly every stage of mast cell biology, including progenitor differentiation, proliferation of immature and mature cells, survival through inhibition of apoptosis, chemotaxis, adhesion, and effector activation, such as degranulation and cytokine release (68, 69). In culture, mast cells derived from mouse or human progenitors grow and mature efficiently in SCF-containing conditions; although IL-3 can support early growth in mouse cells, SCF strongly enhances both proliferation and maturation (68). SCF can also stimulate the proliferation of fully differentiated mast cells, a property less common in many other hematopoietic lineages (68, 69). At the signaling level, SCF/c-Kit activates multiple pathways, including MAPK/ERK, PI3K/AKT, p38, and JAK/STAT, with particularly robust ERK activation contributing to mast cell survival, expansion, and cytokine production (70, 71). In human skin mast cells, phosphoproteomic studies have shown that SCF induces broad phosphorylation changes, with ERK emerging as a central functional pathway (70). SCF also cooperates with FcϵRI, IL-33, and danger-associated signals, such as ATP, to amplify mast cell cytokine responses, thereby integrating growth and survival cues with effector activation (68, 72, 73). Although SCF is essential for tissue mast cells, one study reported that early human mast cell progenitors can still be detected and can proliferate and mature *in vitro* without SCF/c-Kit signaling. This suggests that SCF-c-Kit is not required for the earliest developmental stages but becomes important later for survival and complete maturation in tissues (74).

SCF, through c-Kit, influences mast cell development, migration, adhesion, and persistence in airway tissues, thereby shaping their tissue homing and positioning within the airway submucosa. SCF acts as a strong chemoattractant for mast cells and their precursors, promoting directional migration of both mucosal and connective tissue mast cells *in vitro* and *in vivo* (75, 76). SCF-induced c-Kit activation converges with α4 integrin signaling through PI3K and Rac to promote integrin-directed migration on fibronectin, suggesting that SCF may coordinate cellular activation with extracellular-matrix interactions (77, 78). SCF can also enhance chemokine responsiveness by enabling CCR2-dependent signaling in mast cell progenitors, thereby promoting CCL2-guided migration and recruitment to inflamed lung tissue (79).

SCF enhances mast cell adhesion to fibronectin in a dose-dependent way through integrin receptors, helping cells maintain stable contact with the submucosal matrix (80); this effect depends on SCF signaling through c-Kit rather than SCF serving as a matrix ligand.

Airway structural cells supply SCF that helps anchor mast cells in specific tissue niches. In asthma, the airway epithelium shows increased SCF and c-Kit expression, which supports the local accumulation of mast cells and other c-Kit^+^ cells (35). Airway smooth muscle bundles also express membrane-bound SCF, and along with CADM1 and IL-6, this supports human lung mast cell survival and proliferation (81, 82). SCF therefore functions both as a long-range attractant and as a local retention and survival factor, coordinating mast cell homing into, and stable positioning within, the airway submucosa and smooth muscle layer.

SCF amplifies IgE/FcϵRI-dependent mast cell activation mainly by lowering the activation threshold and enhancing downstream signaling, rather than triggering strong degranulation on its own. This co-stimulation is highly relevant in allergic inflammation, where SCF is elevated in tissues. In human mast cells, SCF acting through c-Kit strongly boosts antigen-induced histamine and leukotriene release, as well as cytokine production such as IL-6, IL-8, IL-13, and GM-CSF, even at antigen levels that would otherwise cause only weak responses (83, 84). In human skin mast cells, SCF preferentially enhances FcϵRI-driven degranulation while suppressing MRGPRX2-mediated pseudo-allergic degranulation, biasing responses toward classical allergic pathways (85). Consistent with this, *in vivo* data show that SCF-rich tissues can support stronger histamine release following IgE receptor cross-linking (86). Mechanistically, SCF-c-Kit and FcϵRI signals converge to optimize mast cell function by engaging overlapping MAPK, PI3K/Akt, and NF-κB pathways, while c-Kit adds strong PI3K and STAT5/6 input (84). These pathways lower the threshold for mast cell activation and promote both rapid secretion and later cytokine gene transcription. Beyond acute degranulation, SCF can also promote the release of mediators such as histamine, TNF-α, and chemokines, including CCL17 and CCL2 (87). Overall, c-Kit acts as a priming receptor that makes mast cells more responsive to FcϵRI stimulation during allergic inflammation (88–90).

SCF through c-Kit can both directly stimulate mast cell mediator release and prime cells for stronger IgE/FcϵRI-dependent activation, with the size and type of response varying by mediator and tissue. SCF can directly induce histamine release, including from mast cells in human skin, lung, uterus, heart, pulmonary tissue, and bone marrow, and this effect is dose-dependent and C-Kit-dependent (83, 91, 92). A short SCF pre-exposure also markedly boosts anti-IgE triggered histamine release from skin, lung, intestinal, and airway mast cells and basophils, effectively lowering the activation threshold (83, 91, 93). SCF also affects lipid mediator production. It can directly trigger PGD_2_ generation in human skin and lung mast cells, and in some settings this response is comparable to anti-IgE stimulation (91, 92). In mouse bone-marrow mast cells, SCF produces an early cysteinyl leukotriene dominant phase followed by a later PGD_2_ dominant phase, while SCF-differentiated human mast cells also release PGD_2_ after FcϵRI cross-linking (94). For cysteinyl leukotrienes, SCF directly promotes LTC_4_ and related mediator production and strongly enhances their release when combined with IgE/FcϵRI signaling, especially in intestinal mast cells. Mechanistically, SCF-c-Kit activates PI3K, PLCγ, and downstream kinase pathways that integrate with FcϵRI signaling to shape both degranulation and lipid mediator synthesis (78, 84, 95, 96). Overall, SCF-c-Kit is best understood as both a direct activator and a potent amplifier of mast cell responses, with particularly strong effects on histamine, PGD_2_, and cysteinyl leukotrienes (histamine, tryptase, PGD_2_, cysteinyl leukotrienes).

### 5.3 Eosinophils

During type 2 inflammation, eosinophil production is controlled by stage specific cytokines. SCF mainly acts on early hematopoietic progenitors and cooperates with IL-3, GM-CSF, and IL-5, whereas IL-5 is the principal lineage selective driver of terminal eosinophil differentiation and survival (97, 98). Reports of c-Kit on mature human eosinophils support functional SCF responses, including adhesion, but c-Kit abundance can vary with tissue source, activation state, and detection method; SCF should therefore not be presented as a universal eosinophil-lineage requirement (99, 100).

In murine eosinophils, SCF activates MEK/ERK- and PI3K-associated pathways and can promote survival, adhesion, chemokine production, leukotriene release, and degranulation (39, 101–104). MEK inhibition blocked SCF-mediated survival in the cited murine study, whereas PI3K inhibition had a smaller effect, supporting an important role for MEK/ERK in that experimental system.

SCF is upregulated in chronic allergic tissues such as the airways and esophagus, where fibroblasts and other structural cells produce high levels of SCF (39, 102, 104). Neutralizing SCF or deleting fibroblast-derived SCF reduces eosinophil accumulation, Th2 cytokines, mucus and fibrosis, and overall allergic pathology, supporting a key *in vivo* role for SCF-driven eosinophil survival and retention (39, 102, 104).

Eosinophils can also produce SCF themselves, which may reinforce local feedback loops that sustain mast cells and other c-Kit^+^ cells in chronic lesions (105, 106). Relative to classic eosinophil survival factors such as IL-5, IL-3, and GM-CSF, SCF adds an IL-5-independent pro-survival pathway that may help explain persistent tissue eosinophilia even when IL-5 is blocked (39, 101–104).

### 5.4 Dendritic cells and basophils

SCF–c-Kit signaling contributes to allergic inflammation by regulating dendritic cell (DC) functions that influence T-cell polarization. In experimental models, allergen-associated stimuli induce c-Kit expression in DCs, supporting IL-6 production and the development of Th2 and Th17 responses. Functional impairment of c-Kit reduces DC expression of Jagged-2, a Notch ligand associated with Th2 differentiation, and attenuates allergic airway inflammation (107). Additional evidence links allergen recognition to c-Kit regulation: TLR2 silencing in cultured mouse DCs reduces house dust mite–induced c-Kit expression, CD80/CD86 expression, and IL-6 production while increasing IL-12 (107, 108). Together, these findings suggest that c-Kit participates in the cytokine and costimulatory programs through which DCs shape allergen-driven adaptive immune responses.

In human basophils, SCF modulates recruitment, survival, and effector responses. IL-3 markedly increases c-Kit expression following 24–48 hours of culture, potentially enhancing basophil responsiveness to SCF. Although SCF alone does not directly induce migration, it amplifies chemotactic responses to eotaxin, MCP-2, MIP-1α, and C5a and enhances integrin upregulation. SCF also cooperates with IL-3 to delay apoptosis and prolong basophil survival, with these effects involving PI3K-dependent signaling (109). Beyond these effects, SCF enhances IgE-dependent histamine release from human peripheral blood basophils, demonstrating its capacity to amplify allergic effector responses (91). Thus, SCF may support basophil participation in allergic inflammation by enhancing recruitment, prolonging survival, and potentiating mediator release.

## 6 Clinical relevance and therapeutic opportunities

### 6.1 Allergic asthma

SCF-c-Kit activity is most extensively studied in allergic asthma. Airway SCF expression and circulating SCF are increased in subsets of patients, and serum concentrations have been associated with disease severity and inhaled-glucocorticoid requirement (110). Experimental blockade of SCF or SCF248 reduces mast cells, eosinophils, ILC2s, type 2 cytokines, mucus-associated responses, airway hyperresponsiveness, and remodeling (23, 39, 106, 111). Evidence in neutrophilic asthma also suggests that fibroblast-derived SCF can augment ILC3-associated IL-17A responses, indicating that the pathway may extend beyond classical type 2 diseases (112). Taken together, these mechanisms emphasize that inhibition of SCF/SCF248 isoform offers a highly selective, upstream therapeutic strategy.

### 6.2 Food allergy and anaphylaxis

In a murine model of established food allergy, selective SCF248 neutralization reduced diarrhea, prevented the challenge-associated fall in body temperature, decreased intestinal permeability, reduced intestinal mast-cell numbers and serum mast-cell protease-1, and lowered antigen-specific IL-4 and IL-5 responses. Treatment also reduced mesenteric lymph node ILC2 numbers and remained protective when administered therapeutically after disease had established. These findings position SCF248 as a regulator of the mast-cell-rich intestinal effector environment that determines the severity of anaphylaxis, while remaining preclinical (113).

### 6.3 Eosinophilic esophagitis

In experimental eosinophilic esophagitis induced with peanut allergen, SCF was increased in esophageal tissue. SCF neutralization reduced esophageal mast cells and eosinophils, decreased local and lymph-node type 2 cytokine responses, lowered peanut-specific IgE, and improved histopathology. Deletion of SCF primarily in collagen-expressing myofibroblasts produced similar reductions, supporting stromal SCF as an important driver of the esophageal inflammatory niche (102, 104). These data broaden the relevance of SCF beyond the airway and show that the pathway can coordinate both eosinophil- and mast-cell-rich mucosal disease.

### 6.4 Therapeutic interpretation

Collectively, asthma, food allergy, and EoE models suggest that targeting inducible SCF248 may suppress several c-Kit^+^ effector populations simultaneously while preserving the constitutive SCF220-dependent homeostatic program. While pan-anti-c-Kit monoclonal antibodies are clinically valuable as short-course conditioning agents for transplantation (114), c-Kit/SCF signaling is indispensable for homeostatic bone marrow function (115). Consequently, systemic receptor blockade disrupts acute erythroid expansion, posing risks of severe anemia (116) that limit its safety profile for long-term maintenance in chronic mucosal diseases. Conversely, selective anti-SCF248 therapy bypasses these systemic safety challenges because the cleavable variant is preferentially overexpressed by structural cells locally at sites of chronic inflammation. Clinical translation of this axis will require isoform-specific pharmacology, careful assessment of localize depletion of inflammatory cells versus systemic hematopoietic effects, and validation in human tissues.

## 7 Conclusions

SCF-c-Kit is a multicellular regulatory axis in allergic disease. SCF248 is inducible in stromal and mesenchymal compartments during inflammation and can amplify mast-cell, eosinophil, and ILC2 responses, whereas SCF220 appears to contribute more consistently to baseline membrane associated signaling. Work from our group has defined complementary roles for this pathway in chronic allergic asthma and ILC2 biology (23, 39, 111, 117), food induced anaphylaxis (113), eosinophilic esophagitis (104), and hematopoietic ILCp-to-ILC2 maturation and mucosal inflammation (23). Together, these studies support SCF248 as a candidate upstream therapeutic target. Future work should define isoform-specific functions in human tissues, establish pharmacodynamic biomarkers, and determine whether selective inhibition of SCF248 can reduce allergic inflammation without disrupting essential c-Kit-dependent homeostasis.

## References

[1] Kolkhir P Akdis CA Akdis M Bachert C Bieber T Canonica GW . Type 2 chronic inflammatory diseases: targets, therapies and unmet needs. Nat Rev Drug Discov. (2023) 22:743–67. doi: 10.1038/s41573-023-00750-1 37528191

[2] Ogulur I Mitamura Y Yazici D Pat Y Ardicli S Li M . Type 2 immunity in allergic diseases. Cell Mol Immunol. (2025) 22:211–42. doi: 10.1038/s41423-025-01261-2 39962262 PMC11868591

[3] Akdis CA Arkwright PD Bruggen MC Busse W Gadina M Guttman-Yassky E . Type 2 immunity in the skin and lungs. Allergy. (2020) 75:1582–605. doi: 10.1111/all.14318 32319104

[4] Rabe KF Rennard S Martinez FJ Celli BR Singh D Papi A . Targeting type 2 inflammation and epithelial alarmins in chronic obstructive pulmonary disease: A biologics outlook. Am J Respir Crit Care Med. (2023) 208:395–405. doi: 10.1164/rccm.202303-0455ci 37348121

[5] Nakayama T Hirahara K Onodera A Endo Y Hosokawa H Shinoda K . Th2 cells in health and disease. Annu Rev Immunol. (2017) 35:53–84. doi: 10.1146/annurev-immunol-051116-052350 27912316

[6] Sugita K Steer CA Martinez-Gonzalez I Altunbulakli C Morita H Castro-Giner F . Type 2 innate lymphoid cells disrupt bronchial epithelial barrier integrity by targeting tight junctions through IL-13 in asthmatic patients. J Allergy Clin Immunol. (2018) 141:300–10:e311. doi: 10.1016/j.jaci.2017.02.038 28392332

[7] Sugita K Altunbulakli C Morita H Sugita A Kubo T Kimura R . Human type 2 innate lymphoid cells disrupt skin keratinocyte tight junction barrier by IL-13. Allergy. (2019) 74:2534–7. doi: 10.1111/all.13935 31166013

[8] Lennartsson J Ronnstrand L . Stem cell factor receptor/c-Kit: from basic science to clinical implications. Physiol Rev. (2012) 92:1619–49. doi: 10.1152/physrev.00046.2011 23073628

[9] Zhou BO Yu H Yue R Zhao Z Rios JJ Naveiras O . Bone marrow adipocytes promote the regeneration of stem cells and haematopoiesis by secreting SCF. Nat Cell Biol. (2017) 19:891–903. doi: 10.1038/ncb3570 28714970 PMC5536858

[10] Galli SJ Zsebo KM Geissler EN . The kit ligand, stem cell factor. Adv Immunol. (1994) 55:1–96. doi: 10.1016/s0065-2776(08)60508-8 7508174

[11] Lev S Blechman JM Givol D Yarden Y . Steel factor and c-kit protooncogene: genetic lessons in signal transduction. Crit Rev Oncog. (1994) 5:141–68. doi: 10.1615/critrevoncog.v5.i2-3.30 7531500

[12] Broudy VC . Stem cell factor and hematopoiesis. Blood. (1997) 90:1345–64. doi: 10.1182/blood.v90.4.1345 9269751

[13] Lemmon MA Ferguson KM . A new twist in the transmembrane signaling tool-kit. Cell. (2007) 130:213–5. doi: 10.1016/j.cell.2007.07.006 17662934

[14] Liu H Chen X Focia PJ He X . Structural basis for stem cell factor-KIT signaling and activation of class III receptor tyrosine kinases. EMBO J. (2007) 26:891–901. doi: 10.1038/sj.emboj.7601545 17255936 PMC1794399

[15] Galli SJ Tsai M Wershil BK . The c-kit receptor, stem cell factor, and mast cells. What each is teaching us about the others. Am J Pathol. (1993) 142:965–74. PMC1886888 7682764

[16] Mcniece IK Briddell RA . Stem cell factor. J Leukoc Biol. (1995) 58:14–22. doi: 10.1002/jlb.58.1.14 7542304

[17] Hsu YR Wu GM Mendiaz EA Syed R Wypych J Toso R . The majority of stem cell factor exists as monomer under physiological conditions. Implications for dimerization mediating biological activity. J Biol Chem. (1997) 272:6406–15. doi: 10.1074/jbc.272.10.6406 9045664

[18] Linenberger ML Jacobson FW Bennett LG Broudy VC Martin FH Abkowitz JL . Stem cell factor production by human marrow stromal fibroblasts. Exp Hematol. (1995) 23:1104–14. 7544739

[19] Kim YK Nakagawa N Nakano K Sulakvelidze I Dolovich J Denburg J . Stem cell factor in nasal polyposis and allergic rhinitis: increased expression by structural cells is suppressed by *in vivo* topical corticosteroids. J Allergy Clin Immunol. (1997) 100:389–99. doi: 10.1016/s0091-6749(97)70254-9 9314353

[20] Miyamoto T Sasaguri Y Sasaguri T Azakami S Yasukawa H Kato S . Expression of stem cell factor in human aortic endothelial and smooth muscle cells. Atherosclerosis. (1997) 129:207–13. doi: 10.1016/s0021-9150(96)06043-1 9105563

[21] Fireman E Kivity S Shahar I Reshef T Mekori YA . Secretion of stem cell factor by alveolar fibroblasts in interstitial lung diseases. Immunol Lett. (1999) 67:229–36. doi: 10.1016/s0165-2478(99)00020-6 10369131

[22] Kassel O Schmidlin F Duvernelle C Gasser B Massard G Frossard N . Human bronchial smooth muscle cells in culture produce stem cell factor. Eur Respir J. (1999) 13:951–4. doi: 10.1034/j.1399-3003.1999.13e04.x 10414388

[23] Asai N Lombardo GK Ocadiz-Ruiz R Gu Y Rasky AJ Garcia DS . Stem cell factor 248 shapes ILC2 transcriptional programs and promotes mucosal inflammation in allergic asthma. Front Immunol. (2026) 17:1843080. doi: 10.3389/fimmu.2026.1843080 42465746 PMC13372909

[24] Toksoz D Zsebo KM Smith KA Hu S Brankow D Suggs SV . Support of human hematopoiesis in long-term bone marrow cultures by murine stromal cells selectively expressing the membrane-bound and secreted forms of the human homolog of the steel gene product, stem cell factor. Proc Natl Acad Sci USA. (1992) 89:7350–4. doi: 10.1073/pnas.89.16.7350 1380155 PMC49707

[25] Williams DA Majumdar MK . Analysis of steel factor (stem cell factor) isoforms in the hematopoietic microenvironment. Stem Cells. (1994) 12:67–74. doi: 10.1203/00006450-199411000-00001 7535148

[26] Kapur R Majumdar M Xiao X Mcandrews-Hill M Schindler K Williams DA . Signaling through the interaction of membrane-restricted stem cell factor and c-kit receptor tyrosine kinase: genetic evidence for a differential role in erythropoiesis. Blood. (1998) 91:879–89. doi: 10.1182/blood.v91.3.879 9446648

[27] Andre C Martin E Cornu F Hu WX Wang XP Galibert F . Genomic organization of the human c-kit gene: evolution of the receptor tyrosine kinase subclass III. Oncogene. (1992) 7:685–91. 1373482

[28] Reilly JT . Class III receptor tyrosine kinases: role in leukaemogenesis. Br J Haematol. (2002) 116:744–57. doi: 10.1046/j.0007-1048.2001.03294.x 11886377

[29] Lemmon MA Pinchasi D Zhou M Lax I Schlessinger J . Kit receptor dimerization is driven by bivalent binding of stem cell factor. J Biol Chem. (1997) 272:6311–7. doi: 10.1074/jbc.272.10.6311 9045650

[30] Ronnstrand L . Signal transduction via the stem cell factor receptor/c-Kit. Cell Mol Life Sci. (2004) 61:2535–48. doi: 10.1007/s00018-004-4189-6 PMC11924424 15526160

[31] Blume-Jensen P Janknecht R Hunter T . The kit receptor promotes cell survival via activation of PI 3-kinase and subsequent Akt-mediated phosphorylation of Bad on Ser136. Curr Biol. (1998) 8:779–82. doi: 10.1016/s0960-9822(98)70302-1 9651683

[32] Lennartsson J Blume-Jensen P Hermanson M Ponten E Carlberg M Ronnstrand L . Phosphorylation of Shc by Src family kinases is necessary for stem cell factor receptor/c-kit mediated activation of the Ras/MAP kinase pathway and c-fos induction. Oncogene. (1999) 18:5546–53. doi: 10.1038/sj.onc.1202929 10523831

[33] Agarwal S Kazi JU Ronnstrand L . Phosphorylation of the activation loop tyrosine 823 in c-Kit is crucial for cell survival and proliferation. J Biol Chem. (2013) 288:22460–8. doi: 10.1074/jbc.m113.474072 23803604 PMC3829335

[34] Lukacs NW Strieter RM Lincoln PM Brownell E Pullen DM Schock HJ . Stem cell factor (c-kit ligand) influences eosinophil recruitment and histamine levels in allergic airway inflammation. J Immunol. (1996) 156:3945–51. doi: 10.4049/jimmunol.156.10.3945 8621935

[35] Al-Muhsen SZ Shablovsky G Olivenstein R Mazer B Hamid Q . The expression of stem cell factor and c-kit receptor in human asthmatic airways. Clin Exp Allergy. (2004) 34:911–6. doi: 10.1111/j.1365-2222.2004.01975.x 15196279

[36] Lei Z Liu G Huang Q Lv M Zu R Zhang GM . SCF and IL-31 rather than IL-17 and BAFF are potential indicators in patients with allergic asthma. Allergy. (2008) 63:327–32. doi: 10.1111/j.1398-9995.2007.01566.x 18053009

[37] Kalmarzi RN Foroutan A Abdi M Ataee P Jalili A Babaei E . Serum level of stem cell factor and its soluble receptor in aspirin-exacerbated respiratory disease. Immunotherapy. (2019) 11:1283–91. doi: 10.2217/imt-2019-0042 31530062

[38] Dolgachev VA Ullenbruch MR Lukacs NW Phan SH . Role of stem cell factor and bone marrow-derived fibroblasts in airway remodeling. Am J Pathol. (2009) 174:390–400. doi: 10.2353/ajpath.2009.080513 19147822 PMC2630549

[39] Fonseca W Rasky AJ Ptaschinski C Morris SH Best SKK Phillips M . Group 2 innate lymphoid cells (ILC2) are regulated by stem cell factor during chronic asthmatic disease. Mucosal Immunol. (2019) 12:445–56. doi: 10.1038/s41385-018-0117-1 30617299 PMC6375742

[40] Hochdorfer T Winkler C Pardali K Mjosberg J . Expression of c-Kit discriminates between two functionally distinct subsets of human type 2 innate lymphoid cells. Eur J Immunol. (2019) 49:884–93. doi: 10.1002/eji.201848006 30892687

[41] Nussbaum JC Van Dyken SJ Von Moltke J Cheng LE Mohapatra A Molofsky AB . Type 2 innate lymphoid cells control eosinophil homeostasis. Nature. (2013) 502:245–8. doi: 10.1038/nature12526 24037376 PMC3795960

[42] Kato A . Group 2 innate lymphoid cells in airway diseases. Chest. (2019) 156:141–9. doi: 10.1016/j.chest.2019.04.101 31082387 PMC7118243

[43] Rodriguez-Rodriguez N Gogoi M Mckenzie ANJ . Group 2 innate lymphoid cells: Team players in regulating asthma. Annu Rev Immunol. (2021) 39:167–98. doi: 10.1146/annurev-immunol-110119-091711 33534604 PMC7614118

[44] Bartemes KR Kita H . Roles of innate lymphoid cells (ILCs) in allergic diseases: The 10-year anniversary for ILC2s. J Allergy Clin Immunol. (2021) 147:1531–47. doi: 10.1016/j.jaci.2021.03.015 33965091 PMC8114584

[45] Mjosberg JM Trifari S Crellin NK Peters CP Van Drunen CM Piet B . Human IL-25- and IL-33-responsive type 2 innate lymphoid cells are defined by expression of CRTH2 and CD161. Nat Immunol. (2011) 12:1055–62. doi: 10.1038/ni.2104 21909091

[46] Chu DK Llop-Guevara A Walker TD Flader K Goncharova S Boudreau JE . IL-33, but not thymic stromal lymphopoietin or IL-25, is central to mite and peanut allergic sensitization. J Allergy Clin Immunol. (2013) 131:187–200:e181-188. doi: 10.1016/j.jaci.2012.08.002 23006545

[47] Kim BS Siracusa MC Saenz SA Noti M Monticelli LA Sonnenberg GF . TSLP elicits IL-33-independent innate lymphoid cell responses to promote skin inflammation. Sci Transl Med. (2013) 5:170ra116. doi: 10.4049/jimmunol.190.supp.114.21 PMC3637661 23363980

[48] Bartemes KR Kephart GM Fox SJ Kita H . Enhanced innate type 2 immune response in peripheral blood from patients with asthma. J Allergy Clin Immunol. (2014) 134:671–8:e674. doi: 10.1016/j.jaci.2014.06.024 25171868 PMC4149890

[49] Doherty TA Scott D Walford HH Khorram N Lund S Baum R . Allergen challenge in allergic rhinitis rapidly induces increased peripheral blood type 2 innate lymphoid cells that express CD84. J Allergy Clin Immunol. (2014) 133:1203–5. doi: 10.1016/j.jaci.2013.12.1086 24582313 PMC3972276

[50] Lao-Araya M Steveling E Scadding GW Durham SR Shamji MH . Seasonal increases in peripheral innate lymphoid type 2 cells are inhibited by subcutaneous grass pollen immunotherapy. J Allergy Clin Immunol. (2014) 134:1193–5:e1194. doi: 10.1016/j.jaci.2014.07.029 25212194

[51] Christianson CA Goplen NP Zafar I Irvin C Good JT Jr. Rollins DR . Persistence of asthma requires multiple feedback circuits involving type 2 innate lymphoid cells and IL-33. J Allergy Clin Immunol. (2015) 136:59–68:e14. doi: 10.1016/j.jaci.2014.11.037 25617223 PMC4494983

[52] Doherty TA Baum R Newbury RO Yang T Dohil R Aquino M . Group 2 innate lymphocytes (ILC2) are enriched in active eosinophilic esophagitis. J Allergy Clin Immunol. (2015) 136:792–4:e793. doi: 10.1016/j.jaci.2015.05.048 26233928 PMC4562810

[53] Poposki JA Klingler AI Tan BK Soroosh P Banie H Lewis G . Group 2 innate lymphoid cells are elevated and activated in chronic rhinosinusitis with nasal polyps. Immun Inflammation Dis. (2017) 5:233–43. doi: 10.1002/iid3.161 28474861 PMC5569375

[54] Imai Y Yasuda K Nagai M Kusakabe M Kubo M Nakanishi K . IL-33-induced atopic dermatitis-like inflammation in mice is mediated by group 2 innate lymphoid cells in concert with basophils. J Invest Dermatol. (2019) 139:2185–94:e2183. doi: 10.1016/j.jid.2019.04.016 31121178

[55] Simoni Y Fehlings M Kloverpris HN Mcgovern N Koo SL Loh CY . Human innate lymphoid cell subsets possess tissue-type based heterogeneity in phenotype and frequency. Immunity. (2017) 46:148–61. doi: 10.1016/j.immuni.2016.11.005 27986455 PMC7612935

[56] Olsthoorn SEM Onrust-Van Schoonhoven A De Bruijn MJW Weekers J Van Nimwegen M Van Beek G . Common gamma-chain cytokines induce an epigenomically plastic precursor-like KIT(+) ILC2 state linked to immune disease susceptibility. J Allergy Clin Immunol. (2026) 158:1117–32. doi: 10.1016/j.jaci.2026.05.029 42309230

[57] Silver JS Kearley J Copenhaver AM Sanden C Mori M Yu L . Inflammatory triggers associated with exacerbations of COPD orchestrate plasticity of group 2 innate lymphoid cells in the lungs. Nat Immunol. (2016) 17:626–35. doi: 10.1038/ni.3443 27111143 PMC5345745

[58] Bal SM Golebski K Spits H . Plasticity of innate lymphoid cell subsets. Nat Rev Immunol. (2020) 20:552–65. doi: 10.1038/s41577-020-0282-9 32107466

[59] Bal SM Bernink JH Nagasawa M Groot J Shikhagaie MM Golebski K . IL-1beta, IL-4 and IL-12 control the fate of group 2 innate lymphoid cells in human airway inflammation in the lungs. Nat Immunol. (2016) 17:636–45. doi: 10.1038/ni.3444 27111145

[60] Golebski K Ros XR Nagasawa M Van Tol S Heesters BA Aglmous H . IL-1beta, IL-23, and TGF-beta drive plasticity of human ILC2s towards IL-17-producing ILCs in nasal inflammation. Nat Commun. (2019) 10:2162. doi: 10.1038/s41467-019-09883-7 31089134 PMC6517442

[61] Alkon N Bauer WM Krausgruber T Goh I Griss J Nguyen V . Single-cell analysis reveals innate lymphoid cell lineage infidelity in atopic dermatitis. J Allergy Clin Immunol. (2022) 149:624–39. doi: 10.1016/j.jaci.2021.07.025 34363841 PMC9130781

[62] Bernink JH Ohne Y Teunissen MBM Wang J Wu J Krabbendam L . c-Kit-positive ILC2s exhibit an ILC3-like signature that may contribute to IL-17-mediated pathologies. Nat Immunol. (2019) 20:992–1003. doi: 10.1038/s41590-019-0423-0 31263279

[63] Ju X Fard NE Bhalla A Dvorkin-Gheva A Xiao M Radford K . A population of c-kit(+) IL-17A(+) ILC2s in sputum from individuals with severe asthma supports ILC2 to ILC3 trans-differentiation. Sci Transl Med. (2025) 17:eado6649. doi: 10.1126/scitranslmed.ado6649 39813318

[64] Mincham KT Loewenthal LE Meyer GFM Fontanella S Marfia M Martin MG . Severe asthma is characterized by a sex-specific ILC landscape and aberrant airway profile that is suppressed by anti-IL-5/5Ralpha biologics. Sci Transl Med. (2025) 17:eadu4913. doi: 10.1126/scitranslmed.adu4913 41032623

[65] Van Der Ploeg EK Golebski K Van Nimwegen M Fergusson JR Heesters BA Martinez-Gonzalez I . Steroid-resistant human inflammatory ILC2s are marked by CD45RO and elevated in type 2 respiratory diseases. Sci Immunol. (2021) 6:eabd3489. doi: 10.1126/sciimmunol.abd3489 33514640

[66] Wada T Yasutomi M Yamada K Hashimura K Kunikata M Tanaka T . Heterogeneity of keratin expression and actin distribution in benign and malignant mammary diseases. Anticancer Res. (1991) 11:1983–93. 1723260

[67] Iemura A Tsai M Ando A Wershil BK Galli SJ . The c-kit ligand, stem cell factor, promotes mast cell survival by suppressing apoptosis. Am J Pathol. (1994) 144:321–8. PMC1887147 7508684

[68] Tsai M Valent P Galli SJ . KIT as a master regulator of the mast cell lineage. J Allergy Clin Immunol. (2022) 149:1845–54. doi: 10.1016/j.jaci.2022.04.012 35469840 PMC9177781

[69] Tsai M Takeishi T Thompson H Langley KE Zsebo KM Metcalfe DD . Induction of mast cell proliferation, maturation, and heparin synthesis by the rat c-kit ligand, stem cell factor. Proc Natl Acad Sci USA. (1991) 88:6382–6. doi: 10.1073/pnas.88.14.6382 1712491 PMC52087

[70] Franke K Kirchner M Mertins P Zuberbier T Babina M . The SCF/KIT axis in human mast cells: Capicua acts as potent KIT repressor and ERK predominates PI3K. Allergy. (2022) 77:3337–49. doi: 10.1111/all.15396 35652819

[71] Teegala LR Elshoweikh Y Gudneppanavar R Thodeti S Pokhrel S Southard E . Protein kinase C alpha and beta compensate for each other to promote stem cell factor-mediated KIT phosphorylation, mast cell viability and proliferation. FASEB J. (2022) 36:e22273. doi: 10.1096/fj.202101838rrr 35349200 PMC9298465

[72] Wershil BK Tsai M Geissler EN Zsebo KM Galli SJ . The rat c-kit ligand, stem cell factor, induces c-kit receptor-dependent mouse mast cell activation *in vivo*. Evidence that signaling through the c-kit receptor can induce expression of cellular function. J Exp Med. (1992) 175:245–55. doi: 10.1084/jem.175.1.245 1370530 PMC2119069

[73] Seifert J Kuchler C Drube S . ATP/IL-33-co-sensing by mast cells (MCs) requires activated c-Kit to ensure effective cytokine responses. Cells. (2023) 12:2696. doi: 10.3390/cells12232696 38067124 PMC10705958

[74] Dahlin JS Ekoff M Grootens J Lof L Amini RM Hagberg H . KIT signaling is dispensable for human mast cell progenitor development. Blood. (2017) 130:1785–94. doi: 10.1182/blood-2017-03-773374 28790106 PMC5659818

[75] Meininger CJ Yano H Rottapel R Bernstein A Zsebo KM Zetter BR . The c-kit receptor ligand functions as a mast cell chemoattractant. Blood. (1992) 79:958–63. doi: 10.1182/blood.v79.4.958.958 1371080

[76] Nilsson G Butterfield JH Nilsson K Siegbahn A . Stem cell factor is a chemotactic factor for human mast cells. J Immunol. (1994) 153:3717–23. doi: 10.4049/jimmunol.153.8.3717 7523504

[77] Tan BL Yazicioglu MN Ingram D Mccarthy J Borneo J Williams DA . Genetic evidence for convergence of c-Kit- and alpha4 integrin-mediated signals on class IA PI-3kinase and the Rac pathway in regulating integrin-directed migration in mast cells. Blood. (2003) 101:4725–32. doi: 10.1182/blood-2002-08-2521 12560232

[78] Reber L Da Silva CA Frossard N . Stem cell factor and its receptor c-Kit as targets for inflammatory diseases. Eur J Pharmacol. (2006) 533:327–40. doi: 10.1016/j.ejphar.2005.12.067 16483568

[79] Collington SJ Hallgren J Pease JE Jones TG Rollins BJ Westwick J . The role of the CCL2/CCR2 axis in mouse mast cell migration *in vitro* and *in vivo*. J Immunol. (2010) 184:6114–23. doi: 10.4049/jimmunol.0904177 20427772 PMC2956277

[80] Dastych J Metcalfe DD . Stem cell factor induces mast cell adhesion to fibronectin. J Immunol. (1994) 152:213–9. doi: 10.4049/jimmunol.152.1.213 7504710

[81] Sutcliffe A Kaur D Page S Woodman L Armour CL Baraket M . Mast cell migration to Th2 stimulated airway smooth muscle from asthmatics. Thorax. (2006) 61:657–62. doi: 10.1136/thx.2005.056770 16601090 PMC2104682

[82] Hollins F Kaur D Yang W Cruse G Saunders R Sutcliffe A . Human airway smooth muscle promotes human lung mast cell survival, proliferation, and constitutive activation: cooperative roles for CADM1, stem cell factor, and IL-6. J Immunol. (2008) 181:2772–80. doi: 10.4049/jimmunol.181.4.2772 18684968 PMC3992374

[83] Bischoff SC Dahinden CA . c-kit ligand: a unique potentiator of mediator release by human lung mast cells. J Exp Med. (1992) 175:237–44. doi: 10.1084/jem.175.1.237 1370529 PMC2119079

[84] Hundley TR Gilfillan AM Tkaczyk C Andrade MV Metcalfe DD Beaven MA . Kit and FcepsilonRI mediate unique and convergent signals for release of inflammatory mediators from human mast cells. Blood. (2004) 104:2410–7. doi: 10.1182/blood-2004-02-0631 15217825

[85] Babina M Guhl S Artuc M Zuberbier T . Allergic FcepsilonRI- and pseudo-allergic MRGPRX2-triggered mast cell activation routes are independent and inversely regulated by SCF. Allergy. (2018) 73:256–60. doi: 10.1111/all.13301 28859248

[86] Hammerberg B Olivry T Orton SM . Skin mast cell histamine release following stem cell factor and high-affinity immunoglobulin E receptor cross-linking in dogs with atopic dermatitis. Vet Dermatol. (2001) 12:339–46. doi: 10.1046/j.0959-4493.2001.00273.x 11844224

[87] Oliveira SH Lukacs NW . Stem cell factor and igE-stimulated murine mast cells produce chemokines (CCL2, CCL17, CCL22) and express chemokine receptors. Inflammation Res. (2001) 50:168–74. doi: 10.1007/s000110050741 11339505

[88] Jensen BM Metcalfe DD Gilfillan AM . Targeting kit activation: a potential therapeutic approach in the treatment of allergic inflammation. Inflammation Allergy Drug Targets. (2007) 6:57–62. doi: 10.2174/187152807780077255 17352689

[89] Draber P Halova I Polakovicova I Kawakami T . Signal transduction and chemotaxis in mast cells. Eur J Pharmacol. (2016) 778:11–23. doi: 10.1016/j.ejphar.2015.02.057 25941081 PMC4630209

[90] Li Y Leung PSC Gershwin ME Song J . New mechanistic advances in FcepsilonRI-mast cell-mediated allergic signaling. Clin Rev Allergy Immunol. (2022) 63:431–46. doi: 10.1007/s12016-022-08955-9 36251242 PMC9575623

[91] Columbo M Horowitz EM Botana LM Macglashan DW Jr. Bochner BS Gillis S . The human recombinant c-kit receptor ligand, rhSCF, induces mediator release from human cutaneous mast cells and enhances IgE-dependent mediator release from both skin mast cells and peripheral blood basophils. J Immunol. (1992) 149:599–608. doi: 10.4049/jimmunol.149.2.599 1378071

[92] Lewis A Wan J Baothman B Monk PN Suvarna SK Peachell PT . Heterogeneity in the responses of human lung mast cells to stem cell factor. Clin Exp Allergy. (2013) 43:50–9. doi: 10.1111/cea.12045 23278880

[93] Bischoff SC Schwengberg S Wordelmann K Weimann A Raab R Manns MP . Effect of c-kit ligand, stem cell factor, on mediator release by human intestinal mast cells isolated from patients with inflammatory bowel disease and controls. Gut. (1996) 38:104–14. doi: 10.1136/gut.38.1.104 8566835 PMC1382987

[94] Mitsui H Furitsu T Dvorak AM Irani AM Schwartz LB Inagaki N . Development of human mast cells from umbilical cord blood cells by recombinant human and murine c-kit ligand. Proc Natl Acad Sci USA. (1993) 90:735–9. doi: 10.1073/pnas.90.2.735 7678463 PMC45740

[95] Murakami M Austen KF Arm JP . The immediate phase of c-kit ligand stimulation of mouse bone marrow-derived mast cells elicits rapid leukotriene C4 generation through posttranslational activation of cytosolic phospholipase A2 and 5-lipoxygenase. J Exp Med. (1995) 182:197–206. doi: 10.1084/jem.182.1.197 7540649 PMC2192097

[96] Nagai S Kitani S Hirai K Takaishi T Nakajima K Kihara H . Pharmacological study of stem-cell-factor-induced mast cell histamine release with kinase inhibitors. Biochem Biophys Res Commun. (1995) 208:576–81. doi: 10.1006/bbrc.1995.1377 7535052

[97] Clutterbuck EJ Hirst EM Sanderson CJ . Human interleukin-5 (IL-5) regulates the production of eosinophils in human bone marrow cultures: comparison and interaction with IL-1, IL-3, IL-6, and GMCSF. Blood. (1989) 73:1504–12. doi: 10.1182/blood.v73.6.1504.1504 2653458

[98] Sonoda Y Arai N Ogawa M . Humoral regulation of eosinophilopoiesis *in vitro*: analysis of the targets of interleukin-3, granulocyte/macrophage colony-stimulating factor (GM-CSF), and interleukin-5. Leukemia. (1989) 3:14–8. 2642572

[99] Robida PA Puzzovio PG Pahima H Levi-Schaffer F Bochner BS . Human eosinophils and mast cells: Birds of a feather flock together. Immunol Rev. (2018) 282:151–67. doi: 10.1111/imr.12638 29431215 PMC5812359

[100] Oliva S Arrigo S Bramuzzo M Cisaro F Dabizzi E Di Nardo G . Eosinophilic esophagitis in children and adolescents: a clinical practice guideline. Ital J Pediatr. (2025) 51:242. doi: 10.1186/s13052-025-02056-x 40702503 PMC12288368

[101] Kobayashi H . Effect of c-kit ligand (stem cell factor) in combination with interleukin-5, granulocyte-macrophage colony-stimulating factor, and interleukin-3, on eosinophil lineage. Int J Hematol. (1993) 58:21–6. 7693027

[102] Oliveira SH Taub DD Nagel J Smith R Hogaboam CM Berlin A . Stem cell factor induces eosinophil activation and degranulation: mediator release and gene array analysis. Blood. (2002) 100:4291–7. doi: 10.1182/blood.v100.13.4291 12453875

[103] Dolgachev V Thomas M Berlin A Lukacs NW . Stem cell factor-mediated activation pathways promote murine eosinophil CCL6 production and survival. J Leukoc Biol. (2007) 81:1111–9. doi: 10.1189/jlb.0906595 17234680

[104] Ptaschinski C Zhu D Fonseca W Lukacs NW . Stem cell factor inhibition reduces Th2 inflammation and cellular infiltration in a mouse model of eosinophilic esophagitis. Mucosal Immunol. (2023) 16:727–39. doi: 10.1016/j.mucimm.2023.07.006 37557983 PMC10680063

[105] Hartman M Piliponsky AM Temkin V Levi-Schaffer F . Human peripheral blood eosinophils express stem cell factor. Blood. (2001) 97:1086–91. doi: 10.1182/blood.v97.4.1086 11159541

[106] Dolgachev V Berlin AA Lukacs NW . Eosinophil activation of fibroblasts from chronic allergen-induced disease utilizes stem cell factor for phenotypic changes. Am J Pathol. (2008) 172:68–76. doi: 10.2353/ajpath.2008.070082 18156208 PMC2189616

[107] Krishnamoorthy N Oriss TB Paglia M Fei M Yarlagadda M Vanhaesebroeck B . Activation of c-Kit in dendritic cells regulates T helper cell differentiation and allergic asthma. Nat Med. (2008) 14:565–73. doi: 10.1038/nm1766 18454155 PMC3664066

[108] Simonetti S Seijas ABB Natalini A Vitale S Runci D Soriani A . Dendritic cells modulate c-kit expression on the edge between activation and death. Eur J Immunol. (2019) 49:534–45. doi: 10.1002/eji.201847683 30758056

[109] Heinemann A Sturm GJ Ofner M Sturm EM Weller C Peskar BA . Stem cell factor stimulates the chemotaxis, integrin upregulation, and survival of human basophils. J Allergy Clin Immunol. (2005) 116:820–6. doi: 10.1016/j.jaci.2005.06.008 16210056

[110] Makowska JS Cieslak M Kowalski ML . Stem cell factor and its soluble receptor (c-kit) in serum of asthmatic patients- correlation with disease severity. BMC Pulm Med. (2009) 9:27. doi: 10.1186/1471-2466-9-27 19480722 PMC2701918

[111] Rasky A Habiel DM Morris S Schaller M Moore BB Phan S . Inhibition of the stem cell factor 248 isoform attenuates the development of pulmonary remodeling disease. Am J Physiol Lung Cell Mol Physiol. (2020) 318:L200–11. doi: 10.1152/ajplung.00114.2019 31747308 PMC6985874

[112] Shao JS Lai AC Huang WC Wu KC Chi PY Chang YM . Pulmonary fibroblast-derived stem cell factor promotes neutrophilic asthma by augmenting IL-17A production from ILC3s. J Clin Invest. (2025) 135:e187372. doi: 10.1172/jci187372 40674143 PMC12352899

[113] Ptaschinski C Rasky AJ Fonseca W Lukacs NW . Stem cell factor neutralization protects from severe anaphylaxis in a murine model of food allergy. Front Immunol. (2021) 12:604192. doi: 10.3389/fimmu.2021.604192 33786039 PMC8005333

[114] Denis M Swartzrock L Willner H Bubb QR Haslett E Chan YY . Hematopoiesis after anti-CD117 monoclonal antibody treatment in the settings of wild-type and Fanconi anemia mice. Haematologica. (2024) 109:2920–9. doi: 10.3324/haematol.2023.284275 38572555 PMC11367201

[115] Karima G Kim HD . Unlocking the regenerative key: Targeting stem cell factors for bone renewal. J Tissue Eng. (2024) 15:20417314241287491. doi: 10.1177/20417314241287491 39479284 PMC11523181

[116] Broudy VC Lin NL Priestley GV Nocka K Wolf NS . Interaction of stem cell factor and its receptor c-kit mediates lodgment and acute expansion of hematopoietic cells in the murine spleen. Blood. (1996) 88:75–81. doi: 10.1182/blood.v88.1.75.bloodjournal88175 8704204

[117] Campbell E Hogaboam C Lincoln P Lukacs NW . Stem cell factor-induced airway hyperreactivity in allergic and normal mice. Am J Pathol. (1999) 154:1259–65. doi: 10.1016/s0002-9440(10)65377-1 10233863 PMC1866576

